# 198. Evaluation of Oral Step-down versus Continued Intravenous Antimicrobial Therapy in Immunocompromised Patients Hospitalized with Gram-Negative Bacteremia

**DOI:** 10.1093/ofid/ofab466.400

**Published:** 2021-12-04

**Authors:** Heather Savage, Catherine H Vu, DeMaurian Mitchner, Amir Zaki

**Affiliations:** 1 University of Louisiana Monroe College of Pharmacy, New Orleans, Louisiana; 2 UF Health Shands, Belleville, New Jersey; 3 University Medical Center New Orleans, New Orleans, LA

## Abstract

**Background:**

Bloodstream infections are associated with considerable morbidity and mortality in the United States. Most patients initially receive parenteral antibiotics for gram-negative bacteremia, and more data is emerging supporting de-escalation to oral (PO) antibiotics to complete treatment. Previous studies evaluating PO antibiotics for gram-negative bacteremia often exclude or have underrepresented immunocompromised patients. This study evaluated clinical failure in immunocompromised patients receiving intravenous (IV) antibiotics compared to patients transitioned to PO antibiotics for gram-negative bacteremia.

**Methods:**

A single center, retrospective cohort study was conducted at 446-bed academic medical center. Patients were included if they were immunocompromised and admitted with a positive blood culture for *E. coli, Klebsiella spp., Citrobacter spp., Serratia spp., Enterobacter spp., Proteus spp., P. aeruginosa*. between November 4, 2017 to November 4, 2020. Patients were excluded from this study if they had polymicrobial bacteremia, no source control within the first 5 days, or an indication for prolonged duration of treatment. The primary endpoint of this study was clinical failure defined as an escalation from PO to IV antibiotics, worsening clinical status, or readmission for the same infection within 30 days of discharge. The secondary endpoints included 30-day mortality, 90-day mortality, 30-day readmission, and time to microbiologic clearance.

**Results:**

A total of 31 immunocompromised patients were included in the study with 26 patients receiving PO step-down therapy and 5 patients being continued on IV treatment for gram-negative bacteremia. There was no difference in the primary outcome of clinical failure between the PO step-down group versus the IV therapy group (15.4% vs 20%; p = 0.613). The most common immunocompromised state in both groups was being HIV positive. Patients in the PO step-down group had a significantly shorter hospital length of stay (7.4 days vs. 13.6 days; p = 0.016).

**Conclusion:**

Oral step-down therapy for gram-negative bacteremia showed similar clinical failure rates to continuous IV therapy in the immunocompromised patient population and may be an option to shorten hospital length of stay.

**Disclosures:**

**All Authors**: No reported disclosures

